# Human Bone Marrow Mesenchymal Stem Cell (hBMSCs)-Derived miR-29a-3p-Containing Exosomes Impede Laryngocarcinoma Cell Malignant Phenotypes by Inhibiting PTEN

**DOI:** 10.1155/2022/8133632

**Published:** 2022-10-18

**Authors:** Tingting Yu, Hong Yu, Dong Xiao, Xiangyan Cui

**Affiliations:** Department of Otolaryngology, Head and Neck Surgery, The First Hospital, Jilin University, Changchun, Jilin, China

## Abstract

Although microRNA-29a-3p was reported to inhibit laryngocarcinoma progression, the potential mechanisms have not been explored clearly. Laryngocarcinoma tissues were collected for analyzing the levels of miR-29a-3p and phosphatase and tensin homolog (PTEN). The miR mimics or inhibitor was transfected into laryngocarcinoma cell lines M4E and Hep2 for the investigation of the biological functions (proliferative, invasion, migratory rates, and apoptotic rates) of this miRNA. The exosomes (Exo) from human bone marrow mesenchymal stem cells (hBMSCs) after the transfection of miR mimics/inhibitor/si-PTEN were isolated and used to stimulate M4E and Hep2 cells. The *in vivo* mouse model was constructed to verify our findings. The miR-29a-3p level was decreased, and PTEN level was elevated in laryngocarcinoma tissues and the cancer cell lines. MiR mimics could inhibit proliferative, invasive migratory rates while promoting apoptotic rates of M4E and Hep2 cells. MiR-29a-3p was enriched in hBMSC-derived Exo, and the Exo from miR-29a-3p mimics transfected hBMSCs could inhibit laryngocarcinoma cell malignant phenotypes *in vitro* and prevent tumor progression *in vivo*. In addition, the direct binding relationship between miR-29a-3p and PTEN in this disease was determined. In conclusion, hBMSC-derived Exo with upregulated miR-29a-3p inhibited laryngocarcinoma progression via regulating PTEN, providing a potential diagnostic and therapeutic target in this disease.

## 1. Introduction

As a common malignant tumor in the upper respiratory tract, laryngocarcinoma accounts for approximately 25 to 30% of cancer cases worldwide [1, 2]. Although several efficient therapies for this disease including surgical operation, and the combination of radiotherapy and chemotherapy, have been developed in recent years, the prognosis of patients at advanced stage is still poor [3]. Therefore, the investigation of the underlying mechanisms during laryngocarcinoma progression may be helpful to find novel treatment strategies in this disease.

microRNAs (miRNAs) have been emerged as a class of novel noncoding RNA regulator, and their length are approximately 22 nucleotides in length [4]. Increasing evidences determined that the occurrence and development of laryngocarcinoma were involved in the dysregulation of a large number of miRNAs. For example, miR-552 promotes cell proliferative rate of laryngocarcinoma cells by modulating p53 signaling [5]. MiR-125b participates in the oncogenic role of lncRNA MALAT1 in the phenotypes including cell proliferative and invasive rates in laryngocarcinoma [6]. MiR-29a-3p, a miRNA, was identified as a tumor-suppressing factor in various human cancers such as endometrial cancer [7], gastric cancer [8], and cervical cancer [9]. Recently, few studies revealed that miR-29a-3p could suppress the proliferative and invasive rates in laryngocarcinoma cells [10, 11]. Although the tumor-suppressive role of this miRNA in laryngocarcinoma is known, its potential mechanisms remain unclear.

Exosome (Exo) (30–200 nm) is a small single-membrane vesicle and exists in almost all mammalian cells [12, 13]. It has been reported that exosomes always act as the cargo carrier for miRNAs and is a promising therapeutic tool [14]. Previous studies indicated that exosomal miR-29a-3p derived from human bone mesenchymal stem cells (hBMSCs) could efficiently suppress cell migrative ability and vasculogenic mimicry in glioma [15]. Due to its role in laryngocarcinoma, we speculated that the exosomal miR-29a-3p derived from hBMSCs might also exert a potential impact in cancer progression.

Phosphatase and tensin homolog (PTEN) is a well-known tumor suppressor protein and has been identified to participate in various tumor progression including prostate cancer [16], osteosarcoma [17], non-small-cell lung cancer [18], and laryngeal carcinomas [19]. Previous studies revealed that inhibition of miR-744-3p could restore PTEN expression, and then suppressed laryngeal squamous cell carcinoma progression [20], suggesting that upregulation of PTEN could contribute to cancer development. More interestingly, PTEN was identified to be one of the downstream genes of miR-29a-3p and participated in the regulation of this miRNA during human diseases [21, 22]. This study aimed to investigate the role of exosomal miR-29a-3p in laryngocarcinoma, as well as the regulatory relationship between miR-29a-3p and PTEN.

In this study, we further verified the downregulation of miR-29a-3p in laryngocarcinoma. Notably, exosomal miR-29a-3p-derived hBMSCs could prevent against the development of this disease, and the impact was involved in the change of PTEN level. The binding relationship between miR-29a-3p and PTEN was also elucidated in our study. Our results provided that hBMSC-derived exosomes might be a potential treatment target for laryngocarcinoma.

## 2. Materials and Methods

### 2.1. Clinical Samples

A total of 30 laryngocarcinoma patients were recruited at the First Hospital of Jilin University, and the tumor tissues as well as the adjacent normal tissues were collected and snap-frozen in liquid nitrogen for the gene expression analysis. All patients have signed the informed consents.

### 2.2. Cell Culture and Transient Transfection

Human bone marrow mesenchymal stem cells (hBMSCs), laryngocarcinoma cell lines SUN-899, M4E, TU212, and the normal pharyngeal epithelial cell line NP69 were obtained from Bank of Chinese Academy of Sciences (Shanghai, China). The Hep-2 cell line was purchased from American type culture collection (ATCC; Manassas, VA, USA). All cell types were grown within Dulbecco's Modified Eagle's Medium (DMEM) with 10% fetal bovine serum (FBS) in a 37°C incubator.

To manipulate the expression levels of miRNA and PTEN in cells, the small interfering RNA for PTEN (si-PTEN) and nonspecifc control (si-NC) were synthesized from Shanghai GenePharma Co., Ltd, and miR mimics/miR-NC and miR inhibitor/inhibitor NC were purchased from Guangzhou Ribobio Co., Ltd. The siRNAs/mimics/inhibitor were transiently transfected into Hep2 and M4E cells under the help of Lipofectamine 2000 (Invitrogen).

### 2.3. Exosome Isolation and Identification

The surface markers of hBMSCs (CD14, CD29, CD34, CD45, D73, CD90, and CD105) were analyzed by flow cytometry. HBMSCs on passage 4-6 were cultured with serum-free medium for 72 h, and the supernatant was centrifuged through differential centrifugation as previously described [23]. After that, the pellet was washed by phosphate buffered saline (PBS) for twice, and the final pellet was collected in PBS through 2 h of ultrahigh speed (100,000 × *g*), that is, hBMSC-derived exosomes (BMSC-Exo). The concentration of BMSC-Exo was determined by BCA kit (Beyotime, China).

The BMSC-derived Exo was visualized under a transmission electron microscopy (TEM; Hitachi-7500; Tokyo, Japan). Meanwhile, the exosomes were lysed by RIPA lysis buffer to extract the Exo protein, and the Exo-specific markers (CD9, CD63, and TSG101) were detected by western blot. To obtain the hBMSC-derived Exo with gene expression change of interest genes, hBMSCs were transfected with siRNA/mimics/inhibitor, followed by the isolation of Exo.

### 2.4. Exosome Treatment

To investigate the impact of hBMSC-derived Exo on biological functions of laryngocarcinoma cell lines, Exo derived from hBMSCs transfected with mimics/inhibitor/siRNAs and named by Exo miR-NC, Exo miR mimic, Exo si-PTEN, Exo si-NC, Exo miR inhibitor, Exo inhibitor NC, or Exo miR inhibitor + si-PTEN. Then, 200 *μ*g hBMSC-Exo (after transfection) or negative control PBS were added into Hep2 and M4E cells. Two days later, cells were harvested for the subsequent function analysis.

### 2.5. Cell Proliferation

Cell counting kit (CCK)-8 (Dojindo, Tokyo, Japan) was used to evaluate cell viabilities as previously reported [24]. Absorbance values at time point of 24, 48, 72, and 96 h were detected at 450 nm. For colony formation, 1 × 10^3^ cells were plated into 6-well plates and cultured for two weeks. Then cells were fixed with methanol for 10 min and stained by 0.1% crystal violet for 15 min. The images were photographed with a light microscope, and colony numbers were counted by naked eyes.

### 2.6. Flow Cytometry

Cells were seeded into 6-well plates with 1 × 10^6^ cells/well and cultured overnight, and the supernatant was removed by centrifugation. By using the Annexin V-FITC cell apoptosis detection kit (Biovision, USA), cell pellets were incubated with 500 *μ*L loading buffer, 5 *μ*L Annexin V-FITC, and 10 *μ*L propidium iodide (PI) solution for 15–20 min. Apoptosis rate was measured using the flow cytometry (BD Biosciences).

### 2.7. Transwell Assay

An 8 *μ*m well size Transwell chamber (Corning, N.Y., USA) was used for Transwell assay. For cell invasion, 100 *μ*L of 50 mg/L Matrigel (1 : 40) was coated into the upper surface of chamber bottom membrane. 100 *μ*L of cell suspension with 2 × 10^5^ cells was added to the upper chamber, and the lower chamber was filled with 600 *μ*L 20% FBS contained DMEM medium. After 24 h, cells were fixed by methanol for 10 min and stained with 0.1% crystal violet for imaging and counting under five random fields. Cell migration assay was performed as same to invasion assay without coating the Matrigel.

### 2.8. Quantitative Reverse Transcription Polymerase Chain Reaction (qRT-PCR)

Total RNA was extracted by TRIzol reagent (Invitrogen), and the cDNAs were synthesized using specific Reverse Transcription Kit (Applied Biosystems). The subsequent PCR reactions were conducted with SYBR green Super-mix (Thermo Fisher) on ABI 7500 PCR fast detection system. The gene expression analysis was evaluated based on the 2^–ΔΔCT^ method. GAPDH/U6 was used as the normalization control. The sequences of primers in this study are as follows: miR-29a-3p, F: 5′ AACAGGTGACTGGTTAGACAA 3′, R: 5′ GTGCAGGGTCCGAGGT 3′; U6, F: 5′ TCGCTTCGGCAGCACATATACT 3′, R: 5′ ACGCTTCACGAATTTGCGTGT 3′; GAPDH, F: 5′ GGCCCAGAATGCAGTTCGCCTT 3′, R: 5′ AATGGCACCCTGCTCACGCA 3′; PTEN, F: 5′ CTTACAGTTGGGCCCTGTACCATCC 3′, R: 5′ TTTGATGCTGCCGGTAAACTCCACT 3′.

### 2.9. Western Blot

Total protein samples were extracted by RIPA buffer. A total of 50 *μ*g protein sample/lane was separated by 10% SDS-PAGE. After transferring into PVDF membranes, the membrane was incubated with the appropriate primary antibodies CD9 (1 : 1000), CD63 (1 : 1000), Tsg101 (1 : 1000), Calnexin (1 : 1000), and PTEN (1 : 1000) overnight at 4°C, followed by the incubation with HRP-conjugated secondary antibody (1 : 10000) for 1 h. All antibodies were purchased from Abcam. The images of protein bands were developed using chemiluminescence reagent, and Image J software was used to gray value analysis.

### 2.10. Luciferase Reporter Assay

The full length of PTEN 3′UTR covering wild-type or mutant miR-29a-3p binding sites were subcloned into pmirGLO dual-luciferase reporter vectors (Promega). After that, the recombinant vectors (PTEN WT and PTEN MUT (the sequence of 3′UTR of PTEN that predicted to interact with miR-29a-3p was mutated into base complementary sequences)) were individually cotransfected into M4E cells with miR mimics or miR-NC. Two days later, the luciferase activity was tested by a dual-luciferase reporter assay system.

### 2.11. RNA Pull-down Assay

PTEN biotin and PTEN nonbiotin labelled probes (Bio-PTEN and Bio-NC) were constructed by Shanghai GenePharma Co., Ltd. and treated with M-280 streptavidin-magnetic beads (Invitrogen) for 2 h at 4°C. Then M4E cells were lysed, and the whole lysates were incubated with probe-coated beads overnight at 4°C. Finally, the RNA was extracted and subjected to qRT-PCR analysis for miR-29a-3p enrichment.

### 2.12. *In Vivo* Model

A total of 20 BALB/c nude mice (4–6 weeks) were used to construct the animal model. Approximately 1 × 10^6^ M4E cells were injected into flank regions of mice via subcutaneous injection as previously described [25]. After the mean tumor volume reaching 100 mm^3^, all mice were randomly divided into four groups: Exo miR-NC, Exo miR-29a-3p mimics, Exo miR-29a-3p inhibitor, and Exo miR-29a-3p mimics/inhibitor. Five mice were in each group. A dose of 200 *μ*g/mouse hBMSC-derived Exo was intravenously injected into mice every two days for a total of ten times. Four weeks later, mice were killed by asphyxiation in a CO_2_ chamber; tumors were collected and weighted. The tumor volume was evaluated by (length × width^2^)/2. Meanwhile, the immunohistochemistry (IHC) assay was performed to assess Ki67 positive cells in tumor tissues using anti-Ki67 primary antibody (1 : 200, Abcam) as previously reported [26]. Slides were observed under a light microscope (Olympus, Tokyo, Japan).

### 2.13. Statistical Analysis

All data were shown as mean ± standard deviation (SD), and each experiment was repeated for three independent times. The differences between groups were analyzed by using the paired Student's *t*-test followed by Tukey's post hoc tests when only two groups were compared, or by one-way analysis of variance (ANOVAs) followed by Dunnett's post hoc tests when more than two groups were compared. Statistical significance was set at *p* < 0.05.

## 3. Results

### 3.1. MiR-29a-3p and PTEN Might Play Crucial Roles in Laryngocarcinoma

**Figure 1:**
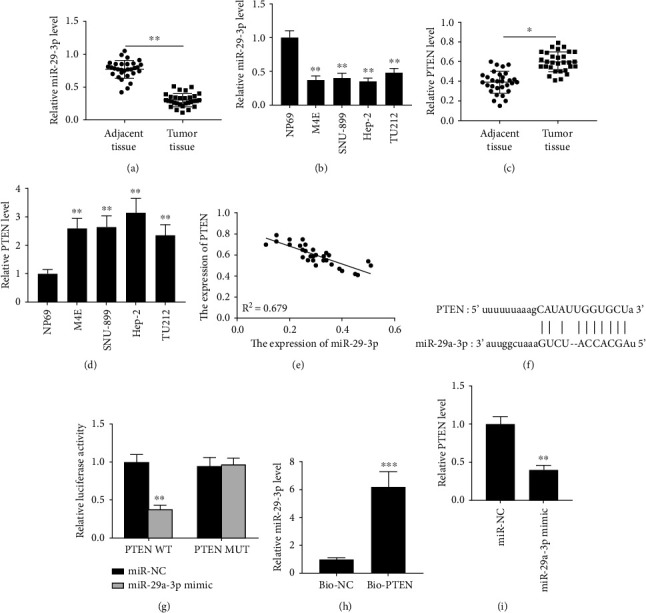
The relationship between miR-29a-3p and PTEN in laryngocarcinoma. (a–b) QRT-PCR analysis of miR-29a-3p in laryngocarcinoma tissues (a) and cancer cell lines (b). (c–d) QRT-PCR analysis of PTEN in laryngocarcinoma tissues (c) and cancer cell lines (d). (e) Correlation analysis between miR-29a-3p and PTEN level in laryngocarcinoma tissues. (f) Base sequence of miR-29a-3p and the wild-type as well as the mutant PTEN 3′UTR by Targetscan. (g) Luciferase activity detection after transfection in M4E cells. (h) The enrichment analysis of miR-29a-3p in M4E cells using biotin labelled PTEN probe. (i) QRT-PCR analysis of PTEN in M4E cells after transfected with miR mimics. ^∗^*p* < 0.05, ^∗∗^*p* < 0.01, and ^∗∗∗^*p* < 0.001.

Firstly, qRT-PCR analysis confirmed that miR-29a-3p level was dramatically decreased in laryngocarcinoma tissues from patients and cancer cell lines (Figures 1(a) and 1(b), all *p* < 0.01). On the contrary, PTEN expression was significantly elevated in tumor tissues and cancer cell lines (Figures 1(c) and 1(d), all *p* < 0.01). The levels of miR-29a-3p and PTEN in tumor tissues were negatively correlated (Figure 1(e), *R*^2^ = 0.679). Although previous studies have reported the binding sites between miR-29a-3p and PTEN 3′UTR sequence (Figure 1(f)), we reverified their interaction. After transfected with miR mimics in M4E cells, the luciferase activity of PTEN WT was significantly reduced, while the PTEN MUT remained unchanged (Figure 1(g), *p* < 0.01). RNA pull-down assay further determined their direct binding M4E cells (Figure 1(h), *p* < 0.001). In addition, after transfected with miR mimic M4E cells, PTEN level was notably reduced compared to miR-NC (Figure 1(i), p < 0.01). It postulates that this axis may play potential roles in laryngocarcinoma.

### 3.2. MiR-29a-3p Upregulation Reduced Laryngocarcinoma Cell Malignant Progression

**Figure 2:**
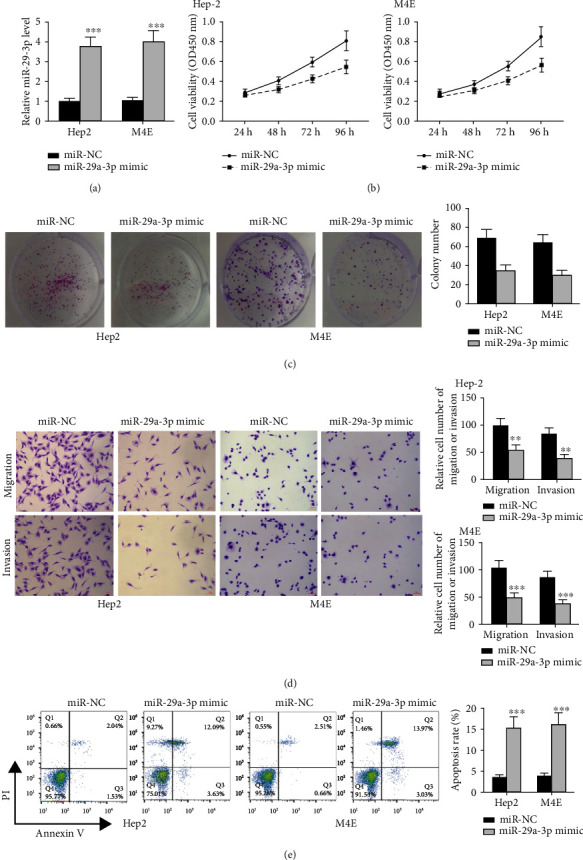
MiR-29a-3p upregulation reduced laryngocarcinoma cell malignant progression. M4E and Hep2 cells were transfected with miR-29a-3p mimics or miR-NC. (a) QRT-PCR analysis of miR-29a-3p. (b) CCK-8 assay. (c) Colony formation assay. (d) Transwell assay for detecting cell invasive and migratory rates. (e) Flow cytometry analysis of apoptotic rate. ^∗^*p* < 0.05, ^∗∗^*p* < 0.01, and ^∗∗∗^*p* < 0.001.

To further confirm its function, we transfected miR mimics into M4E and Hep2 cell lines, and the level of miR-29a-3p was determined by qRT-PCR analysis (Figure 2(a), both *p* < 0.001). As shown in Figures 2(b)–2(e) , we observed that the transfection of miR mimics notably reduced cell proliferative, invasive, and migratory rates while enhanced cell apoptotic rate of two cell lines compared to miR-NC (cell viability, both *p* < 0.05; colony number, both *p* < 0.01; cell invasion and cell migration, all *p* < 0.01; cell apoptosis, both *p* < 0.001). It suggests that miR-29a-3p upregulation can inhibit laryngocarcinoma cell malignant progression *in vitro*.

### 3.3. Identification of hBMSC-Derived Exosomes

**Figure 3:**
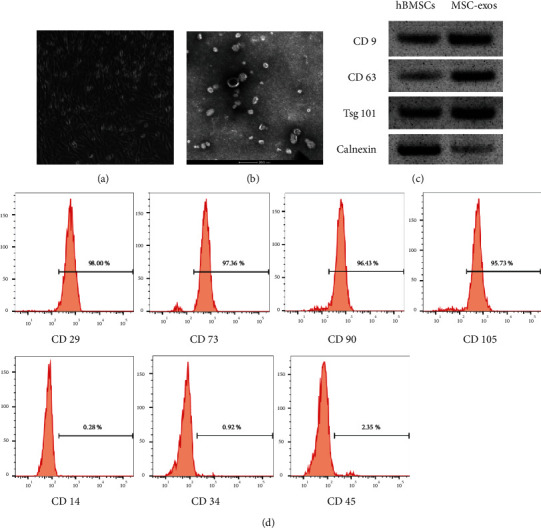
Identification of hBMSC-derived exosomes. (a) The morphology of hBMSCs by inverted microscope. Magnification × 400. (b) The ultrastructure of hBMSC-derived Exo by a TEM (scale bar = 200 nm). (c) Western blot analysis of Exo surface markers. (d) Flow cytometry analysis of hBMSCs' surface markers.

To isolate the exosomes from hBMSCs, we observed the morphology of hBMSCs (Figure 3(a)) and hBMSC-derived Exo (Figure 3(b)). Meanwhile, western blot analysis revealed that Exo surface makers CD9, CD63, and Tsg101 exhibited a relative high level, while Calnexin level showed a low level in BMSC-Exo than that in hBMSCs (Figure 3(c)). In addition, flow cytometry analysis indicated that hBMSCs' surface makers CD29 (98%), CD73 (97.36%), CD90 (96.43%), and CD105 (95.73%) were highly expressed, while CD14 (0.28%), CD34 (0.92%), and CD45 (2.35%) were barely expressed (Figure 3(d)). The data suggest that hBMSC-derived Exo was successfully isolated and can be used for the subsequent studies.

### 3.4. HBMSC-Derived Exo Reduced Laryngocarcinoma Cell Malignant Progression

**Figure 4:**
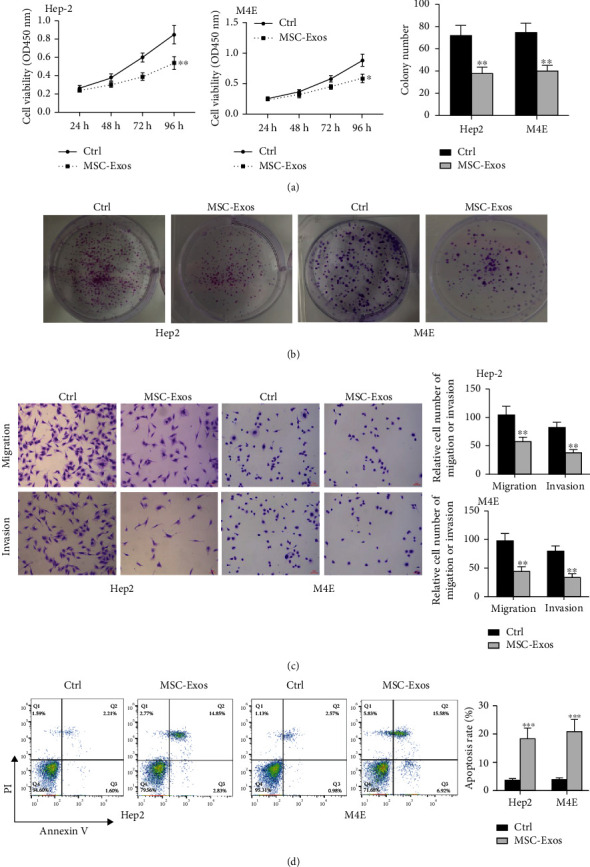
HBMSC-derived Exo reduced laryngocarcinoma cell malignant progression. M4E and Hep2 cells were treated with hBMSC-derived Exo for 48 h. (a) CCK-8 assay. (b) Colony formation assay. (c) Transwell assay for detecting cell invasive and migratory rates. (d) Flow cytometry analysis of apoptotic rate. ^∗^*p* < 0.05, ^∗∗^*p* < 0.01, and ^∗∗∗^*p* < 0.001.

Subsequently, we isolated hBMSC-derived Exo, then stimulated M4E and Hep2 cells with PBS as the control. As shown in Figures 4(a)–4(d), the proliferative, invasive, and migratory rates of two cell lines were significantly suppressed, while cell apoptotic rate was oppositely enhanced by hBMSC-derived Exo compared to control group (cell viability: Hep-2, *p* < 0.01, M4E, *p* < 0.05; colony number, both *p* < 0.01; cell invasion and migration, all *p* < 0.01; cell apoptosis, both *p* < 0.001). Based on these results, we think that hBMSC-derived Exo can attenuate laryngocarcinoma cell progression.

### 3.5. HBMSC-Derived Exo with Upregulated miR-29a-3p Inhibited Laryngocarcinoma Cell Malignant Progression

**Figure 5:**
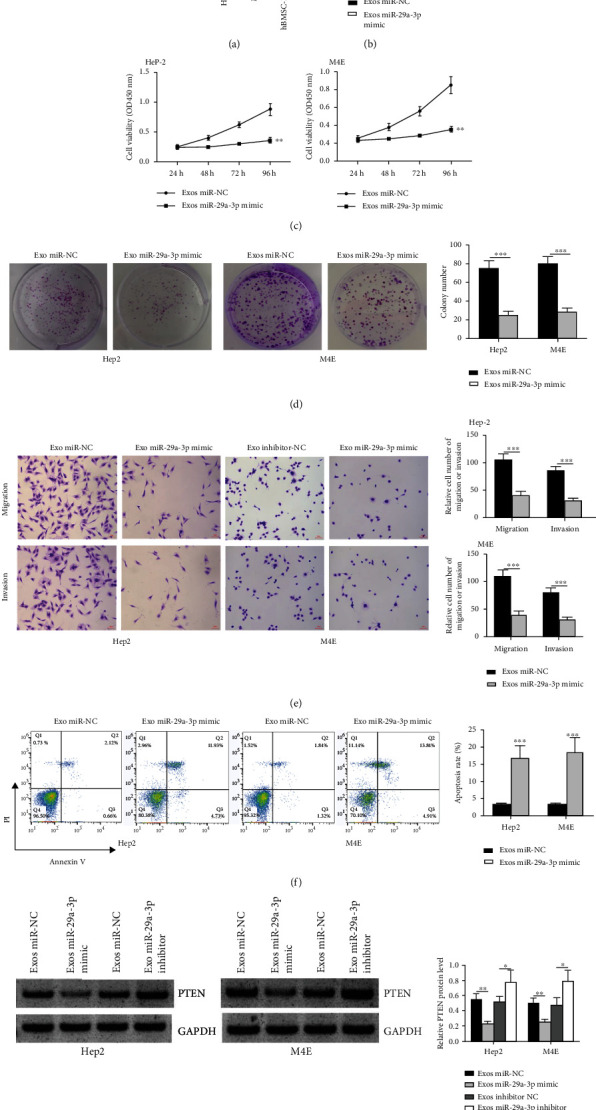
HBMSC-derived Exo with upregulated miR-29a-3p inhibited laryngocarcinoma cell malignant progression. (a) QRT-PCR analysis of miR-29a-3p in hBMSC-derived Exo, M4E, and Hep2 cells. (b) QRT-PCR analysis of miR-29a-3p in hBMSC-derived Exo after the transfection of miR mimics and miR-NC. (c–f) The Exo derived from miR mimics or miR-NC-transfected hBMSCs were used to stimulate M4E and Hep2 cells for 48 h. (c) CCK-8 assay. (d) Colony formation assay. (e) Transwell assay for detecting cell invasive and migratory rates. (f) Flow cytometry analysis of apoptotic rate. (g) Western blot analysis of PTEN in two cell types. ^∗^*p* < 0.05, ^∗∗^*p* < 0.01, and ^∗∗∗^*p* < 0.001.

Interestingly, we observed that miR-29a-3p level in hBMSC-derived Exo was notably higher than that in M4E and Hep2 cells (Figure 5(a), both *p* < 0.001). Then we introduced miR-29a-3p mimics into hBMSCs and isolated miR mimics transfected hBMSC-derived Exo to stimulate M4E and Hep2 cells. MiR-29a-3p level in miR mimics transfected hBMSC-derived Exo was significantly higher than that in miR-NC transfected hBMSC-derived Exo (Figure 5(b), p < 0.001). As shown in Figures 5(c)–5(f), we found that Exo miR-29a-3p mimics notably reduced the proliferative, invasive, and migratory rates of two cell lines, while it enhanced the apoptotic rate of two cell types compared to Exo miR-NC (cell viability, both *p* < 0.01; colony number, both *p* < 0.001; cell invasion and migration, all *p* < 0.001; cell apoptosis, both *p* < 0.001). Exo miR-29a-3p mimics reduced PTEN expression compared with Exo miR-NC, and Exo miR-29a-3p inhibitor enhanced PTEN expression in comparison to Exo inhibitor NC (Figure 5(g), mimic, both *p* < 0.01; inhibitor, both *p* < 0.05). These data indicate that the inhibition of hBMSC-derived Exo on laryngocarcinoma cell malignant progression is associated with the upregulation of miR-29a-3p.

### 3.6. HBMSC-Derived Exo with Downregulated miR-29a-3p Enhanced, and Downregulated PTEN Inhibited Laryngocarcinoma Cell Progression

**Figure 6:**
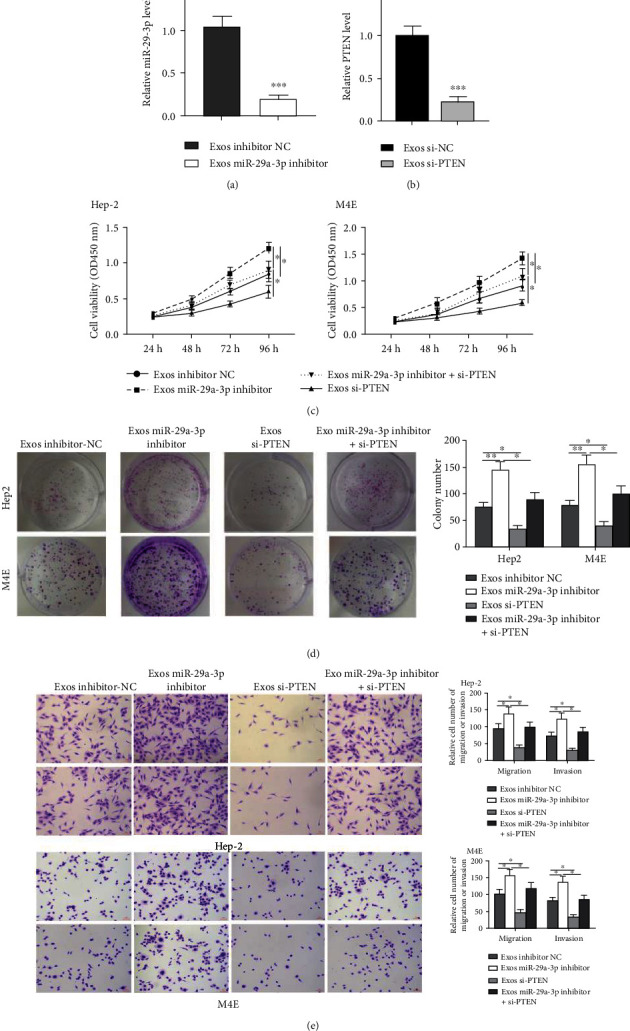
The impacts of Exo miR-29a-3p inhibitor and Exo si-PTEN in laryngocarcinoma cell progression. (a–b) MiR-29a-3p inhibitor and si-PTEN were transfected into hBMSCs, and the Exo was isolated. QRT-PCR analysis of the levels of miR-29a-3p (a) and PTEN (b). (c–f) The Exo derived from inhibitor NC, miR inhibitor, si-PTEN, or miR inhibitor + si-PTEN transfected hBMSCs were used to stimulate M4E and Hep2 cells for 48 h. (c) CCK-8 assay. (d) Colony formation assay. (e) Transwell assay for detecting cell invasive and migratory rates. ^∗^*p* < 0.05, ^∗∗^*p* < 0.01, and ^∗∗∗^*p* < 0.001.

To confirm our findings, we cointroduced miR inhibitor and si-PTEN into hBMSCs and isolated hBMSC-derived Exo. The levels of miR-29a-3p and PTEN in Exo miR-29a-3p inhibitor and Exo si-PTEN were both reduced compared to that in Exo inhibitor NC and Exo si-NC, respectively (Figures 6(a) and 6(b), both *p* < 0.001). As shown in Figures 6(c)–6(e), Exo miR-29a-3p inhibitor enhanced the proliferative, invasive, and migratory rates of two cell lines (cell viability, both *p* < 0.05; colony number, both *p* < 0.01; cell invasion and migration, all *p* < 0.05); Exo si-PTEN reduced the proliferative, invasive, and migratory rates of two cell lines (cell viability, both *p* < 0.05; colony number, both *p* < 0.05; cell invasion and migration, all *p* < 0.05), while cotreatment of Exo miR-29a-3p inhibitor and Exo si-PTEN notably attenuated the impacts of Exo miR-29a-3p inhibitor on these cell malignant phenotypes (cell viability, both *p* < 0.05; colony number, both *p* < 0.05; cell invasion and migration, all *p* < 0.05). These data suggest that exosomal miR-29a-3p from hBMSCs inhibits the laryngocarcinoma progression via PTEN.

### 3.7. HBMSC-Derived Exo with Upregulated miR-29a-3p Inhibited Tumor Growth, and That with Downregulated miR-29a-3p Enhanced Tumor Growth *In Vivo*

**Figure 7:**
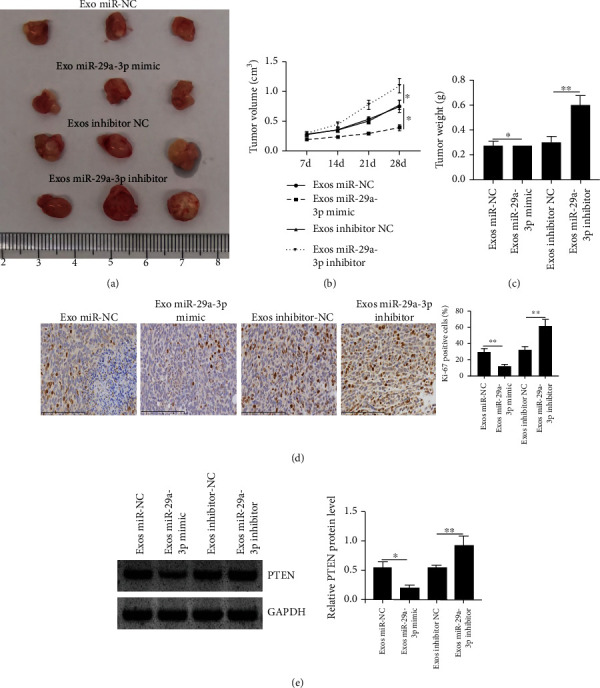
The impacts of Exo miR mimics and Exo inhibitor in tumor progression *in vivo*. (a) Representative images of tumors. (b) Tumor volume. (c) Tumor weight. (d) IHC assay using anti-Ki67 antibody in tumor tissues. Scale bar = 100 *μ*m. (e) Western blot analysis of PTEN in tumor tissues. ^∗^*p* < 0.05 and ^∗∗^*p* < 0.01.

Finally, the *in vivo* animal model was constructed to verify our findings. As shown in Figures 7(a)–7(c), we found that Exo miR-29a-3p mimics inhibited tumor growth, tumor volume, and weight (all *p* < 0.05), while Exo miR-29a-3p inhibitor enhanced tumor progression (all *p* < 0.01). From Ki67 staining results, the number of Ki67 positive cells in tumor tissues was significantly reduced by Exo miR-29a-3p mimics and elevated by Exo miR-29a-3p inhibitor (Figure 7(d), all *p* < 0.01). In addition, western blot analysis showed that Exo miR-29a-3p mimics decreased PTEN level (*p* < 0.01), and Exo miR-29a-3p inhibitor increased PTEN level in tumor tissues (*p* < 0.05) (Figure 7(e)). It indicates that hBMSC-derived Exo can inhibit laryngocarcinoma progression via exosomal miR-29a-3p mediated suppression of PTEN.

## 4. Discussion

In the past decades, stimulating literatures have pointed out the potential of stem cell-derived exosomes in the treatment for different human cancers [27, 28]. Despite of these important significances, more molecular mechanisms involved in exosome treatment have not been well studied. In this study, we revealed that hBMSC-derived exosomes could efficiently inhibit laryngocarcinoma cell malignant phenotypes *in vitro* and tumor growth *in vivo*. Moreover, our data demonstrated that miR-29a-3p and PTEN regulation might account for the suppressive role of hBMSC-derived exosomes in laryngocarcinoma. This study contributed us to understand the potential action mechanism of stem cell-derived exosomes in cancer progression and also develop exosome therapeutic strategy. How to deliver exosomes with changed miRNA level to body and how to make it work without no side effect is becoming a big challenge in laryngocarcinoma.

MiR-29a-3p was observed to be downregulated in many human cancers and thought as a potential tumor-suppressive factor [29]. MiR-29a-3p inhibited the proliferative, migratory, and invasive rates of endometrial cancer cells [7]. Downregulated miR-29a-3p might account for the tumor promotion of the lncRNA KCNQ1OT1 in hepatocellular carcinoma [30]. In addition, miR-29a-3p was also shown to inhibit the proliferative ability of prolactinoma cells via modulating the *β*-catenin signaling pathway [31]. Although the oncogenic role of this miRNA in laryngocarcinoma has been reported, its deliver from hBMSCs and the complex mechanisms remains unclear. Here, we found that miR-29a-3p was significantly enriched in hBMSC-derived exosomes. HBMSC-derived exosomes and hBMSC-derived exosomes with upregulated miR-29a-3p could both inhibit laryngocarcinoma cell malignant phenotypes. The results *in vivo* animal model were keeping with that *in vitro* cell experiments. The hBMSC-derived exosomes carrying miRNA mimics might be a novel therapeutic strategy for laryngocarcinoma and other miR-29a-3p downregulated human cancers.

PTEN is a tumor-related gene and was identified to be associated with the process of multiple malignant tumors including prostate cancer [32], acute myeloid leukemia [33], papillary thyroid cancer [34], and also laryngocarcinoma [35]. A series of previous studies indicated that PTEN was a downstream mRNA target of miR-29a-3p and participated in the regulation of this miRNA in tumor progression. For example, miR-29a-3p regulated the development and progression of abdominal aortic aneurysm via direct interaction with PTEN [21]. MiR-29-3p also regulated the progression of hepatocellular carcinoma through targeting PTEN followed by the NF-*κ*B signaling pathway [36]. However, there were few related reports about the regulation of miR-29a-3p and PTEN in the development of laryngocarcinoma. Our study revealed the binding relationship between miR-29a-3p and PTEN, and further confirmed that this axis closely impacted the progression of laryngocarcinoma by performing the rescue experiments. As we known, one miRNA might target one or multiple downstream mRNAs simultaneously in eukaryotic cells. Except for PTEN, whether there were other potential target mRNAs that participated in regulation of miR-29a-3p in laryngocarcinoma, which should be well investigated in the subsequent experiments. Interestingly, several potential targets of miR-29a-3p have been reported in previous studies including STX17 [37], COL4A1 [38], CCNT2 [39], HDAC4 [40], and so on. We will explore these potential mechanisms in the future.

The PTEN/PI3K/AKT axis is reported to implicate in diverse physiological and pathological conditions and plays an important role in the regulation of cell growth and apoptosis in diverse human cancer including prostate cancer, hepatocellular carcinoma, and pancreatic cancer [41–44]. As the key target of PTEN, PTEN is identified to negatively regulate PI3K/AKT signaling [45, 46]. Based on the regulation relationship between PTEN and PI3K/AKT signaling, we speculated that the effect of miR-2a-3p/PTEN regulation axis in laryngocarcinoma might be also mediated by PI3K/AKT signaling. This needed to be further investigated in the subsequent experiments.

## 5. Conclusion

In summary, our findings thought that hBMSC-derived exosomes could inhibit laryngocarcinoma progression via exosomal miR-29a-3p-mediated suppression of PTEN, suggesting that exosome therapy might be a promising choice for this disease.

## Data Availability

The data that support the findings of this study are available from the corresponding author upon reasonable request.

## Abbreviations

Exo: Exosomes
hBMSCs: Human bone marrow mesenchymal stem cells
miRNAs: microRNAs
PTEN: Phosphatase and tensin homolog
PI: Propidium iodide
SD: Standard deviation.
